# Assessment of arm, neck and shoulder complaints and scapular static malposition among computer users

**DOI:** 10.5327/Z1679443520190329

**Published:** 2019-12-01

**Authors:** Aline Mendonça Turci, Camila Gorla, Michelli Belotti Bersanetti

**Affiliations:** 1 Department of Biological and Health Sciences, Universidade de Araraquara - Araraquara (SP), Brazil. Department of Biological and Health Sciences Universidade de Araraquara Brazil

**Keywords:** questionnaire, workers, musculoskeletal pain

## Abstract

**Background:**

Musculoskeletal complaints of the arm, neck and/or shoulder not attributed to acute trauma or any systemic disorder (CANS) are characterized by symptoms such as pain, numbness and paresthesia which may reach severe and disabling levels and thus significantly interfere with the performance of work and daily living activities. Computer use at work considerably increased in recent years, being attended with a substantial elevation of the prevalence of CANS among individuals who use computers at work.

**Objective:**

To investigate biomechanical and psychosocial risk factors, scapular static imbalance and functional impact on work and daily living activities of upper limb complaints among workers who use computers.

**Methods:**

We analyzed ergonomic and psychosocial risk factors by means of MUEQ-Br, scapular static malposition with the SICK-scapula protocol, and functional impairment in work and daily living activities with DASH. The sample comprised 109 employees of a private institution who use computers at work.

**Results:**

The average scores on body posture and control over tasks were significantly higher among the symptomatic participants. Scapular malposition did not differ between the symptomatic and asymptomatic participants, but functional impairment did.

**Conclusion:**

Awkward posture at work and poor control over tasks seem to contribute to the occurrence of CANS among office workers who use computers. Scapular malposition is not systematically present among individuals with CANS, but the opposite is true. Individuals with CANS exhibited functional impairments.

## RESUMO |

**Introdução:**

Queixas musculoesqueléticas nas regiões dos membros superiores, do complexo do ombro e da cervical não atribuídas a trauma agudo ou a desordens sistêmicas (c*omplaints of arms, neck and shoulders* - CANS) são caracterizadas por sintomas como dor, dormência e parestesia e podem atingir níveis severos e debilitantes, comprometendo o desempenho de atividades de vida diária e ocupacionais. Nos últimos anos, o uso de computador no trabalho intensificou-se, gerando considerável aumento das ocorrências das CANS entre os usuários.

**Objetivo:**

O objetivo foi descrever os fatores de risco biomecânicos e psicossociais, o desequilíbrio estático escapular e o impacto funcional nas atividades ocupacionais e de vida diária decorrente de sinais e sintomas dos membros superiores em trabalhadores usuários de computador.

**Métodos:**

Foram analisados os fatores de risco ergonômicos e psicossociais pelo Maastricht Upper Extremity Questionnaire (MUEQ-Br), o desequilíbrio postural estático da escápula por meio do Scapular Malposition, Inferior Medial Border Prominence, Coracoid Pain and Malposition, and Dyskinesis of Scapular Movement (SICK-Scapula) e o comprometimento funcional das atividades ocupacionais e de vida diária pelo Disabilities of the Arm, Shoulder and Hand (DASH) de 109 trabalhadores usuários de computador do setor privado.

**Resultados:**

Foi encontrada pontuação média significativamente maior para postura e controle de trabalho nos indivíduos sintomáticos. Posicionamento escapular não se mostrou diferente entre as amostras com e sem dor, entretanto foi diferente para impacto funcional.

**Conclusões:**

A adoção de posturas inadequadas no trabalho e o mau gerenciamento das condições de controle de trabalho parecem contribuir para o desenvolvimento das CANS em trabalhadores de escritório que utilizam computador. O desalinhamento estático da escápula não necessariamente está presente em indivíduos com CANS; o inverso é verdadeiro. Ademais, os indivíduos com CANS apresentaram maior impacto funcional.

## INTRODUCTION

The number of people with jobs involving use of computers considerably grew in the past 20 years^1^^,^^2^. In 2000, 60% of workers used computers at work, 80% of them every day^3^. This higher rate is due to the substantial economic development that took place in recent decades, which led to the implementation of computer-based technologies at organizations to improve productivity^1^. However, these developments were not free from impact on the well-being of workers. In a review study, Wahlström^4^ found that 10 to 62% of the involved workers exhibited musculoskeletal disorders.

Complaints of the arm, neck and/or shoulder (CANS) is defined as musculoskeletal complaints of arm, neck and/or shoulder not caused by acute trauma or by any systemic disease^5^. It is characterized by symptoms such as pain, numbness and paresthesia, however, it might reach very severe and disabling levels and thus significantly interfere with the performance of work and daily living activities^6^^,^^7^.

The aforementioned increase in the use of computers at work^1^^,^^2^ was attended by a considerable elevation of the frequency of CANS^1^^,^^7^ in both developed and developing countries^1^^,^^7^. The prevalence of musculoskeletal complaints involving the upper limb, neck and lower back among workers who use computers varies from 20 to 77%^3^, and mainly affect the neck and shoulder^4^^,^^6^.

Kinetic-functional imbalance of the shoulder girdle derived from intermuscular dynamic imbalance, might manifest as static malposition, dynamic abnormalities of the scapulohumeral rhythm, restricted range of motion and shortened length of the shoulder girdle muscles, all of which increase the risk of musculoskeletal injury^8^.

The term SICK scapula (Scapular malposition, Inferior medial border prominence, Coracoid pain and malposition, and dysKinesis of scapular movement) is used refer to injuries resulting from scapular malposition. This condition might be due to muscle insufficiency and imbalance liable to cause depression, protraction and upward rotation of the scapula^9^^,^^10^^,^^11^. While scapular malposition concerns the static posture, its dynamic effects manifest as scapular dyskinesis, which in turn impairs the glenohumeral, acromioclavicular and scapulothoracic joint kinematics^9^^,^^10^^,^^11^.

Musculoskeletal complaints among workers who use computers seem to have multifactorial etiology^4^^,^^6^. Some of the main causes include awkward posture and bad habits in the workplace, the design of workstations and psychosocial factors at work ^4^^,^^12^^,^^13^.

The method most widely used to investigate the influence of physical, ergonomic and psychosocial factors on musculoskeletal disorders is based on symptom reports obtained through questionnaires and interviews^14^^,^^15^. These techniques are particularly advantageous because they allow analyzing psychosocial aspects from the respondents’ perspective, are financially feasible and rapid, whereby they enable large-scale surveys such as those performed in epidemiological studies^16^.

The Maastricht Upper Extremity Questionnaire (MUEQ)^6^ is the single tool available to assess ergonomic and psychosocial aspects among computer users and was specifically designed to characterize CANS in detail^6^. In turn, functional abnormalities of the upper limbs can be assessed by means of the Disabilities of the Arm, Shoulder and Hand (DASH) questionnaire^17^.

The aim of the present study was to investigate biomechanical and psychosocial risk factors, scapular static malposition and functional impacts of upper limb complaints on work and daily living activities among workers who use computers.

## METHODS

### SAMPLING

**Figure 1.**
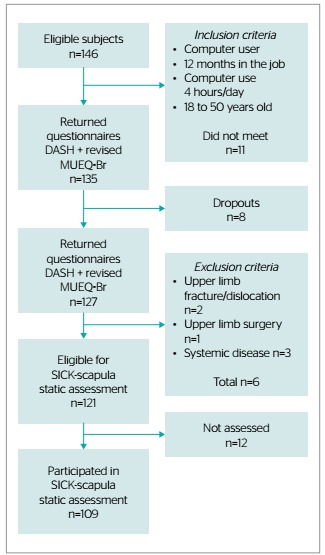
Flowchart represent the sample eligibility, Araraquara, São Paulo, Brazil, 2018 (n=146).

The study population was composed of 146 workers in the private sector who use computers at work. As per the inclusion criteria, we administered MUEQ and DASH to 135 participants; 127 participants returned the questionnaires duly responded, but six were excluded from analysis as per the exclusion criteria. As a result, 121 participants were considered for static scapular assessment, but only 109 attended the scheduled appointments (Figure 1).

Inclusion criteria were: 12 months at least in the current job and work involving computers for at least four uninterrupted hours/day^1^. The study was divulgated in visits to the workplace.

Individuals with cognitive impairment, illiterate, with visual impairment not corrected by glasses, hearing loss not corrected by hearing aids, diseases associated with intellectual impairment, history of fractures involving the shoulder girdle or elbow, dislocation or past surgery of the shoulder or elbow, or systemic diseases such as fibromyalgia, systemic lupus erythematosus and rheumatoid arthritis were excluded.

The present study was approved by the research ethics committee of University of Araraquara, ruling no. 99305/2013, in compliance with the National Health Council Resolution no. 466/2012. All the participants signed an informed consent form.

### ASSESSMENT OF ERGONOMIC AND PSYCHOSOCIAL RISK FACTORS

We administered the MUEQ version translated and validated for use in Brazil (revised MUEQ-Br)^18^. This questionnaire comprises 41 items distributed across six domains-workstations, body posture, control over tasks, job demands, breaks and social support-and comprises one further domain to characterize CANS. Maximum domain scores are: workstation (6 items on aims and physical space)-6; body posture domain (6 items on body position)-18; control over tasks (9 items on self-management)-27; job demands (7 items on work-related pressure-21; breaks (6 items on breaks’ duration)-18; and social support (7 items on work routine)-21.

### ASSESSMENT OF STATIC SCAPULAR MALPOSITION

The aim of SICK scapula assessment is to establish the severity of scapular dysfunction (dyskinesis) based on objective and subjective clinical evidence^9^. Therefore, it includes subjective symptoms, such as pain in definite shoulder sites (periscapular, proximal lateral arm, radicular, coracoid and acromioclavicular joint), objective signs (scapular assistance test, impingement test, paresthesia, coracoid and acromioclavicular pain) and scapular malposition.

Scapular malposition was assessed based on three measurements established in Burkhart et al.’s^9^ protocol, to wit, infera, lateral displacement and abduction (protraction). We considered the spine as reference point, and measurements were made based on the palpation of bone structures, such as the superomedial and inferior angles of the scapula. Infera represents the difference (in centimeters) between the vertical height of the superiomedial angle of each scapula. Scapular lateral displacement is the difference (in centimeters) in the horizontal distance of each scapular superiomedial angle to the spine. In turn, scapular abduction is the difference (in angular degrees, measured with a goniometer) between the medial scapular margins from plumb midline between the SICK and contralateral scapula.

The global score ranges from 0 (healthy symmetrical asymptomatic scapula) to 20 (worst SICK malpositioned scapula with all the attending objective and subjective signs).

SICK scapula assessment was performed by one single blinded examiner. The reference points used were the superior and inferior angles of the scapula and the spinous process of T1 and T2.

### ASSESSMENT OF THE DEGREE OF FUNCTIONAL IMPAIRMENT IN WORK AND DAILY LIVING ACTIVITIES

DASH^17^ was administered to assess the participants’ degree of functional impairment due to upper limb musculoskeletal symptoms and dysfunction^19^. This instrument comprises 21 items to assess activities of daily living, five items to characterize symptoms (pain and weakness), four items to establish the impact of limitations on work, social life and self-image, and eight optional items to evaluate the degree of impairment for work and practice of sports/performing arts. Each item is attributed a score from 1 to 5. Item scores are added and transformed into a 0-100 scale, in which the higher the score, the higher the impact of pain on the respondent’s life. We did not consider the sports/performing arts domain in the present study.

### DATA ANALYSIS

The collected data were subjected to descriptive statistical analysis; variables were described in terms of means, standard deviation (SD) and 95% confidence interval. Data with normal distribution were analyzed with Student’s t-test, and those without normal distribution with the nonparametric Mann-Whitney test. The significance level was set to 0.05.

## RESULTS

A total of 127 out of 146 eligible subjects responded the questionnaires (response rate: 87%) but upon considering the number of effectively assessed participants (n=102) the participation rate decreased to 75%.

Fifty out of the 109 analyzed participants reported some upper limb complaint. The average age of the sample was 34.28 years old (SD=10.93). Seventy participants were female, with average age 33.93 (SD=11.58) and 39 were male, average age 34.90 (SD=9.76). Symptoms were found among 31 women, average age 32.18 (SD=11.29), and 19 men, average age 36 (SD=10.15). Other analyzed characteristics of the participants are described in Table 1. Among the symptomatic participants, the prevalence of CANS at least once weekly in the past three months was 64% (n=32). Pain became chronic among 67.7% of the women and 57.9% of the men.

**Table 1.** Sample characterization according to sex and symptoms, Araraquara, São Paulo, Brazil, 2018 (n=146).

	Asymptomatic	Symptomatic
Full sample		
n=109 Age 34.28 years old (SD 10.93) YWC: 12.12 (SD 7.81) DCUT: 7.68 hours (SD 1.56)	n=59 (54.13%) Age 35.14 years old (SD 10.91) YWC: 12.46 years old (SD 7.96) DCUT: 7.77 hours (SD 1.67)	n=50 (45.87%) Age 33.52 years old (SD 10.98) YWC: 11.43 years old (SD 7.60) DCUT: 7.61 hours (SD 1.48)
Men		
n=39 Age 34.90 years old (SD 9.76) YWC: 13.77 (SD 6.60) DCUT: 7.91 hours (SD 1.59)	n=20 (SD 51.28%) Age 34.74 years old (SD 9.46) YWC: 13.47 (SD 6.87) DCUT: 7.84 hours (SD 1.46)	n=19 (SD 48.71%) Age 36.00 years old (SD 10.15) YWC: 14.05 (SD 6.50) DCUT: 7.97 hours (SD 1.74)
Women		
n=70 Age 33.93 years old (SD 11.58) YWC: 11.20 (SD 8.31) DCUT: 7.56 hours (SD 1.55)	n=39 (55.71%) Age 36.00 years old (SD 11.75) YWC: 11.84 (SD 8.57) DCUT: 7.70 hours (SD 1.73)	n=31 (44.28%) Age 32.18 years old (SD 11.29) YWC: 9.82 (SD 7.92) DCUT: 7.45 hours (SD 1.39)

YWC: years of work with computers; DCUT: daily computer use time; n: sample size; SD: standard deviation; *values expressed as means and standard deviation.

The most frequent musculoskeletal complaint for the entire sample was neck pain (above 44%), followed by shoulder (36%), wrist (12%) and arm (8%) complaints (Table 2). These rates differed on analysis per sex: females - neck (46%), shoulder (26%), arm (19%) and wrist (9%) pain; males-shoulder (42%), neck (31%), wrist (16%) and arm (11%) pain (Table 2).

**Table 2.** Frequency of worst arm, neck and/or shoulder complaints according to the revised Brazilian version of the Maastricht Upper Extremity Questionnaire (MUEQ-Br) among the all the symptomatic participants and distributed per sex, Araraquara, São Paulo, Brazil, 2018 (n=146).

Worst complain site	Total number of symptomatic subjects	Total frequency	Symptomatic women	Frequency (females)	Symptomatic men)	Frequency (males)
Neck	22	44%	14	46%	6	31%
Shoulder	18	36%	8	26%	8	42%
Wrist	6	12%	3	9%	3	16%
Arm	4	8%	6	19%	2	11%
Upper limb	50		31		19	

Relative to the entire sample (symptomatic and asymptomatic participants) we found statistically significant difference on the MUEQ-Br body posture domain, control over tasks and global scores; all three DASH domains (symptoms and activities of daily living, physical ability and occupational activities); and SICK scapula subjective pain, objective pain and global scores (Table 3). On analysis of the symptomatic participants per sex we did not find statistically significant difference relative to MUEQ-Br domains or global score or DASH domains, while differences were found for all SICK-scapula domain (subjective pain, objective pain and scapular malposition) and global score (Table 3).

**Table 3.** (A) Revised Brazilian version of the Maastricht Upper Extremity Questionnaire (MUEQ-Br) global and domain scores for the full sample and compared according to sex and symptoms; (B) Disabilities of the Arm, Shoulder and Hand (DASH) global and domain scores and compared according to sex and symptoms; (C) Scapular malposition, Inferior medial border prominence, Coracoid pain and malposition, and dysKinesis of scapular movement (SICK scapula) global and domain scores and compared according to sex and symptoms, Araraquara, São Paulo, Brazil, 2018 (n=146).

Domain scores		Mean (95%CI)				
Domains	Maximum score	Total n=109	Symptomatic participants n=50	Asymptomatic participants n=59	Symptomatic women n=31	Symptomatic men n=19
(A)						
Workstation	6	0.91 (0.72-1.10)	1.04 (0.73-1.35)	0.78 (0.55-1.01)	1.00 (0.63-1.37)	1.11 (0.55-1.66)
Body posture	18	5.98 (5.33-6.63)	7.44 (6.53-8.35)*	4.59 (3.78-5.41)*	7.58 (6.34-8.82)	7.21 (5.88-8.54)
Control over tasks	27	5.02 (4.22-5.82)	5.80 (4.64-6.96)*	4.15 (3.13-5.18)*	5.97 (4.59-7.34)	5.53 (3.43-7.63)
Job demands	21	3.57 (2.88-4.26)	4.32 (3.13-5.51)	3.29 (2.48-4.10)	4.35 (2.75-5.96)	4.26 (2.48-6.04)
Breaks	18	4.39 (3.77-5.02)	4.30 (3.35-5.25)	4.56 (3.77-5.34)	4.61 (3.30-5.93)	3.79 (2.50-5.08)
Social support	21	2.46 (1.88-3.04)	3.18 (2.10-4.26)	1.98 (1.45-2.51)	3.23 (1.76-4.69)	3.11 (1.52-4.69)
Global score	112	22.57 (20.27-24.86)	26.29 (22.60-29.98)*	19.82 (17.24-22.40)*	27.10 (22.03-32.17)	25.00 (19.78-30.22)
(B)						
Symptoms and ADL	100	10.89 (8.09-13.69)	17.57 (11.54-23.59)*	5.68 (3.57-7.79)*	18.06 (12.39-23.74)	16.75 (3.62-29.89)
Physical ability (sports/performing arts	100	4.83 (2.51-7.15)	7.75 (3.46-12.04)*	2.33 (0.28-4.38)*	9.07 (2.70-15.45)	5.59 (1.11-10.07)
Work	100	8.42 (5.63-11.21)	11.00 (6.78-15.22)*	4.98 (1.64-8.31)*	13.10 (6.98-19.23)	7.57 (2.92-12.21)
(C)						
Subjective pain	5	1.03 (0.83-1.23)	1.42 (1.10-1.74)*	0.69 (0.47-0.92)*	1.74 (1.34-2.15)†	0.89 (0.45-1.34)†
Objective pain	6	1.64 (1.32-1.96)	2.16 (1.65-2.67)*	1.20 (0.84-1.57)*	2.61 (2.01-3.22)†	1.42 (0.58-2.26)†
Scapular malposition	9	1.83 (1.60-2.07)	1.88 (1.51-2.25)	1.80 (1.49-2.11)	1.61 (1.10-2.12)†	2.32 (1.86-2.77)†
Global score	20	4.50 (4.00-5.01)	5.46 (4.67-6.25)*	3.69 (3.10-4.29)*	5.97 (5.00-6.93)†	4.63 (3.33-5.93)†

*Statistically significant difference between symptomatic and asymptomatic participants (p≤0.05); †statistically significant difference according to sex (p≤0.05); 95%CI: 95% confidence interval; ADL: activities of daily living.

## DISCUSSION

Sitting over long periods of time is considered a risk factor for several health problems^20^. The prevalence of CANS was higher among the men (48%) compared to the women (44%), while other studies reported the opposite situation^6^^,^^7^^,^^18^^,^^21^. Complaints became chronic among 64% of the symptomatic participants; this rate was higher for the women (67.74%) compared to the men (57.90%). According to the literature, pain becomes chronic in about 14% of workers who use computers^22^.

Our results indicate that most participants with chronic pain did not discontinue their work, but more than half of them reported some impairment in efficacy in association with pain^23^. One reason for pain to become chronic depends on motor variations in the joints and the whole body. Such variability becomes more substantial when individuals feel pain and might increase the odds for pain to become chronic. It should be noticed that women are more susceptible to these factors than men^24^.

Structural differences between males and females are well known in the literature^24^. Anthropometric data indicate that women are shorter, on average, than men, exhibit larger discrepancy in the length of the legs and wider hips^24^. To this, one should add cultural differences in pain perception and tolerance between the sexes^25^.

The highest prevalence of complaints corresponded to the neck and shoulder, both in regard to the total sample and according to sex. These findings agree with those reported by other authors^1^^,^^6^^,^^7^^,^^18^^,^^21^. Differently, in the study by Ranasinghe e al.^1^ the highest prevalence corresponded to the forearm and hand. Eltayeb et al.^6^ consider that some risk factors are more associated to neck complaints, while others to the wrist and hand. Individuals with shoulder, arm and hand complaints often also complain of neck pain^1^. This topic is still controversial in the literature. According to some reports, the muscle activity demanded by computer work is more strongly associated with arm and hand than with neck and shoulder complaints^18^.

We found higher average scores on the MUEQ posture domain among the symptomatic versus the asymptomatic participants, which points to a relationship between awkward body posture during computer use and CANS. In support of this possibility, previous studies evidenced correlation between musculoskeletal symptoms among office workers and upper limb postural inadequacy at work^26^. The literature calls the attention to the relevance of providing ergonomic training^27^, instead of merely changing the furniture, to reduce ergonomic risks, since sitting over long periods of time is associated with several health problems^28^^,^^29^.

The average score on MUEQ domain control over tasks was significantly higher among the symptomatic participants. This finding suggests that these workers develop poorer ways to perform their tasks by comparison to their decision-making authority and skill development.

To summarize, body posture and control over tasks seem to play a crucial role in the development or perpetuation of work-related musculoskeletal disorders. Agreeing with our results, Gawke et al.^30^ found that aspects related to control over tasks, such as task interdependence and information processing, were reliable predictors of CANS among office workers in the Netherlands. In turn, the analyzed physical aspects of work did not exhibit correlation with occurrence of CANS in the analyzed sample when the independent variables - psychological and social factors - where jointly analyzed in a logistic regression model. As a result, these authors suggest that part of the influence attributed to physical/ergonomic factors on occurrence of CANS might actually be related to psychosocial aspects^30^. Similar results were obtained in the study by Turci et al.^18^, in which poor body posture and control over tasks were more frequent among the computer users with CANS.

Yet, symptoms might have a relationship with the job tasks at the analyzed private institution, even though our data show that their functional impact on activities of daily living was related to pain. Thus being, our results point to the need to include extra-occupational aspects in ergonomic evaluation to thus address the multidimensional nature of human beings. This, however, is mere speculation for the time being, and additional studies are needed to substantiate this assumption.

Scapular malposition did not differ between the symptomatic and asymptomatic participants. SICK-scapula evaluation is a well-known method of static scapular assessment, but that does not provide any information on dynamic aspects whatsoever. On these grounds, one might infer that scapular malposition is not necessarily a cause of pain.

One of the limitations of the present study derives from the sample size. Therefore we recommend conducting studies with larger samples, also in other settings in the private sector, to confirm our results. Then, we did not consider several psychosocial aspects, such as pain catastrophizing, anxiety and depression, beliefs, fear and avoidance of pain, and quality of life. Future studies with larger samples might contribute to a more thorough understanding of the relationship between ergonomic, postural and psychosocial factors involved in the occurrence of CANS among office workers who use computers.

## CONCLUSION

The results of the present study suggest that awkward body posture and poor control over tasks might contribute to the occurrence of upper limb and neck pain among office workers who use computers. Scapular static malposition is not systematically present among individuals with CANS, but the opposite is true. Individuals with CANS exhibited greater functional impairments in work and daily living activities and their physical skills. However, we did not find any difference between men and women with upper limb and neck musculoskeletal pain.
